# The Flavonoid Metabolite 2,4,6-Trihydroxybenzoic Acid Is a CDK Inhibitor and an Anti-Proliferative Agent: A Potential Role in Cancer Prevention

**DOI:** 10.3390/cancers11030427

**Published:** 2019-03-26

**Authors:** Ranjini Sankaranarayanan, Chaitanya K. Valiveti, D. Ramesh Kumar, Severine Van slambrouck, Siddharth S. Kesharwani, Teresa Seefeldt, Joy Scaria, Hemachand Tummala, G. Jayarama Bhat

**Affiliations:** 1 Department of Pharmaceutical Sciences and Translational Cancer Research Center, College of Pharmacy and Allied Health Professions, South Dakota State University, Brookings, SD 57007, USA; ranjini.sankaranarayanan@sdstate.edu (R.S.); chaitanya.valiveti@sdstate.edu (C.K.V.); siddharth.kesharwani@sdstate.edu (S.S.K.); teresa.seefeldt@sdstate.edu (T.S.); hemachand.tummala@sdstate.edu (H.T.); 2 Current Address: Department of Entomology, University of Kentucky, Lexington, KY 40546, USA; rameshinsilico@gmail.com; 3 Department of Chemistry and Biochemistry, South Dakota State University, Brookings, SD 57007, USA; severinevs@gmail.com; 4 Department of Veterinary and Biomedical Sciences, South Dakota State University, Brookings, SD 57007, USA; joy.scaria@sdstate.edu

**Keywords:** colorectal cancer, cyclins, CDKs and CDK inhibitors, flavonoids and polyphenols

## Abstract

Flavonoids have emerged as promising compounds capable of preventing colorectal cancer (CRC) due to their anti-oxidant and anti-inflammatory properties. It is hypothesized that the metabolites of flavonoids are primarily responsible for the observed anti-cancer effects owing to the unstable nature of the parent compounds and their degradation by colonic microflora. In this study, we investigated the ability of one metabolite, 2,4,6-trihydroxybenzoic acid (2,4,6-THBA) to inhibit Cyclin Dependent Kinase (CDK) activity and cancer cell proliferation. Using in vitro kinase assays, we demonstrated that 2,4,6-THBA dose-dependently inhibited CDKs 1, 2 and 4 and in silico studies identified key amino acids involved in these interactions. Interestingly, no significant CDK inhibition was observed with the structurally related compounds 3,4,5-trihydroxybenzoic acid (3,4,5-THBA) and phloroglucinol, suggesting that orientation of the functional groups and specific amino acid interactions may play a role in inhibition. We showed that cellular uptake of 2,4,6-THBA required the expression of functional SLC5A8, a monocarboxylic acid transporter. Consistent with this, in cells expressing functional SLC5A8, 2,4,6-THBA induced CDK inhibitory proteins p21^Cip1^ and p27^Kip1^ and inhibited cell proliferation. These findings, for the first time, suggest that the flavonoid metabolite 2,4,6-THBA may mediate its effects through a CDK- and SLC5A8-dependent pathway contributing to the prevention of CRC.

## 1. Introduction

**Figure 1:**
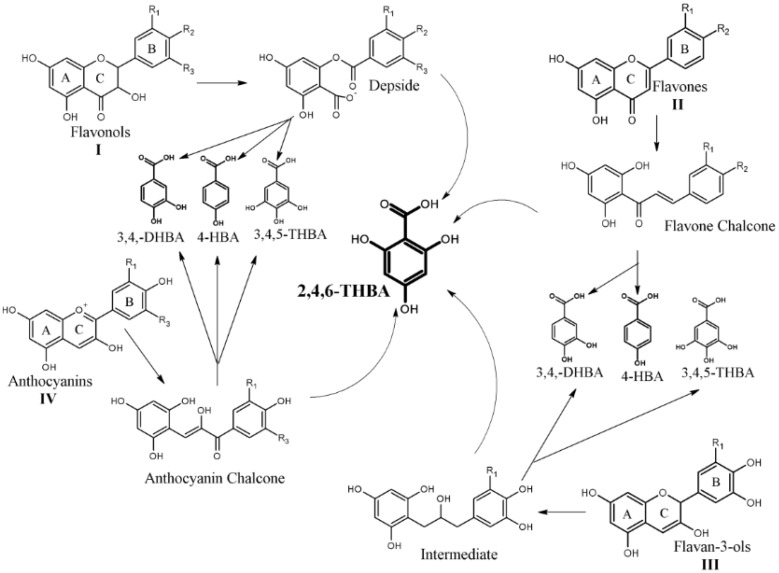
Postulated degradation pathways of selected flavonoid compounds to generate 2,4,6-THBA, 4-HBA, 3,4-DHBA and 3,4,5-THBA. The parent flavonoid compounds (labelled with roman numerals) undergo autodegradation or microbial degradation to generate unstable intermediates such as chalcones or depsides which then further degrade to more stable phenolic acids such as 4-HBA, 3,4-DHBA, 3,4,5-THBA and 2,4,6-THBA. The chemical structure of the flavonoid A-ring (labelled within each flavonoid structure) is conserved and generates 2,4,6-THBA. The flavonoid B-ring (labelled within each flavonoid structure) can generate 4-HBA, 3,4-DHBA and 3,4,5-THBA depending on the number of hydroxyl groups. R_1_, R_2_ and R_3_ represent either hydrogen or hydroxyl groups and based on the position and number of –OH groups in the B-rings flavonoids generate monohydroxy-, dihydroxy- or trihydroxybenzoic acids [9,17,18].

More than one million people are diagnosed with colorectal cancer (CRC) worldwide every year, and it is the third most common cause of cancer death in the United States [1,2]. Studies conducted over the past two decades have clearly established chemo-preventive roles for flavonoids against CRC [3,4,5,6,7]. Though these studies mainly focused on the therapeutic potential and activity of the parent compounds, limited reports exist on the therapeutic role for their metabolites [8,9]. The backbone of flavonoids comprises of an aromatic ring A bound to a heterocyclic ring C, which is in turn attached to a third aromatic ring B through a carbon-carbon bond (Figure 1) [6,9]. They are further classified into anthocyanins, flavonols, flavan-3-ols, flavones, flavanones and isoflavones based on the individual functional groups appended or attached to this backbone. The hydroxylation pattern in the A-ring is mostly conserved whereas the B-ring can have 1 to 3 hydroxyl groups [6]. Members of the flavonoid family are highly unstable and are known to undergo light, temperature and pH dependent degradation [10,11]; the number and position of –OH groups are inversely correlated with stability [12]. Flavonoids are reported to be more stable in acidic conditions (pH < 4); however, at neutral or alkaline pH (pH > 8, as in the intestine), they undergo auto-degradation which generates simpler phenolic acids [13]. Additionally, owing to their low absorption (1–15%) in the small intestine, flavonoids undergo degradation in the colon due to the action of gut microbiota [9,14,15]. The flavonoids absorbed into the systemic circulation may also revert back to the small intestine as conjugated metabolites secreted in the bile fluid, eventually reaching the colonic microflora [8]. These metabolites may undergo de-conjugation and get re-exposed to the gastrointestinal (GI) epithelial cells. Therefore, it is likely that colonic stem/epithelial cells are exposed to these metabolites at relatively high concentrations. In fact, it is reported that phenolic acid concentrations obtained from dietary sources in human fecal matter is relatively high [16].

Following degradation, different flavonoids give rise to different phenolic acids [14]. For example, the B-ring generates 3,4,5-trihydroxybenzoic acid (3,4,5-THBA/gallic acid) from delphinidin with three –OH groups, 3,4-dihydroxybenzoic acid (3,4-DHBA/protocatechuic acid) from cyanidin with two –OH groups, and 4-hydroxybenzoic acid (4-HBA) from pelargonidin with one –OH group [11,19]. The degradation of other flavonoids such as catechin and epigallocatechin gallate also produce these phenolic acids [20,21]. The A-ring from most flavonoids generate an unstable and reactive 2,4,6-trihydroxybenzaldehyde (2,4,6-THBAld) [22,23], which is later oxidized to 2,4,6-trihydroxybenzoic acid (2,4,6-THBA) facilitated by host enzymes in the colon [9]. Due to the reported low bioavailability of flavonoids and their highly unstable nature which results in the generation of phenolic acids, it is suggested that the observed anti-cancer effects of flavonoids may actually come from the degraded products rather than the parent compounds [9,19,22]. This is further substantiated by several reports demonstrating the ability of 3,4-DHBA and 3,4,5-THBA to reduce cancer cell growth [24,25,26,27]. However, limited studies exist regarding the potential of the A-ring derivative 2,4,6-THBA to act as an anti-cancer agent, which is the focus of this study.

Two important observations previously reported by us on 2,4,6-THBA led to the present study. First, while determining the ability of salicylic acid derivatives (2,3-dihydroxybenzoic acid and 2,5-dihydroxybenzoic acid) to inhibit Cyclin Dependent Kinase (CDK) enzyme activity, we observed that 2,4,6-THBA also inhibited its activity in vitro [28]. Secondly, among all the hydroxybenzoic acids tested in that report, 2,4,6-THBA exhibited the highest level of CDK inhibition which prompted us to further examine its potential to inhibit cancer cell growth. CDKs are often dysregulated and overexpressed in cancers and therefore are attractive targets for cancer therapy [29]. The present investigation was carried out to study: (1) the effect of 2,4,6-THBA on CDKs 1, 2 and 4; (2) the interactions of 2,4,6-THBA with CDKs though in silico analysis; (3) to identify a transporter for its cellular uptake; and (4) its ability to inhibit cell proliferation in colon cancer cell lines. In this study we demonstrate that 2,4,6-THBA dose-dependently inhibits activities of CDKs 1, 2 and 4, and identify the specific amino acids involved in these interactions. We show that SLC5A8, a member of the monocarboxylic acid transporter (MCT) family, is required for the cellular uptake of 2,4,6-THBA. In cell lines ectopically expressing SLC5A8, 2,4,6-THBA induced the expression of CDK inhibitors p21^Cip1^ and p27^Kip1^ (mRNA and protein) and this was associated with decreased cell proliferation. Interestingly, the other two metabolites, 3,4-DHBA and 3,4,5-THBA, which failed to inhibit CDK activity, potently inhibited cancer cell proliferation independent of a functional SLC5A8. These results suggest that the flavonoid metabolite 2,4,6-THBA may mediate its anti-cancer effects through a CDK- and SLC5A8-dependent pathway, whereas, 3,4-DHBA and 3,4,5-THBA are likely to exert their effect through a CDK- and SLC5A8-independent pathway.

## 2. Results

### 2.1. Dose Dependent Effect of 2,4,6-THBA on CDK 1, 2 and 4 Enzyme Activity

**Figure 2:**
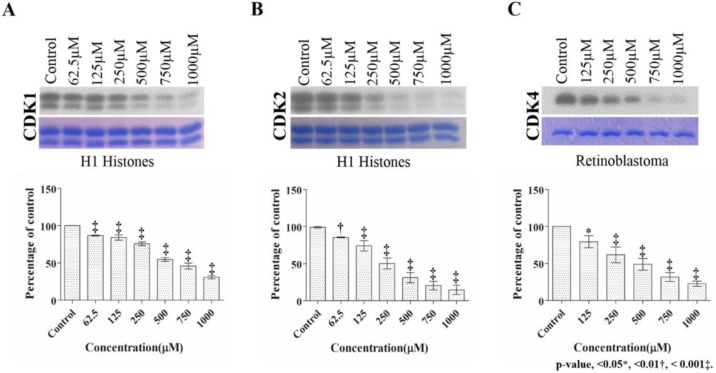
(**A**–**C**) show the dose dependent effects of 2,4,6-THBA on CDK1, CDK2 and CDK4 enzyme activity respectively. H1 histones were used as the substrate for CDK1 and CDK2, while retinoblastoma protein was used as the substrate for CDK4. Upper panel shows phosphorylation of H1 histones (CDK1 and CDK2) and retinoblastoma protein (CDK4) in the presence of different concentrations of 2,4,6-THBA. The middle panel shows the Coomassie Blue stained proteins. Lower panel represents the quantification of the bands in upper panel.

In a previous report, we showed that 2,4,6-THBA inhibits CDK1 activity in vitro; however, a detailed study of its effects on other CDKs or its biological implications was not investigated [28]. We, therefore, initiated this study by analyzing the dose-dependent inhibitory effect of 2,4,6-THBA on recombinant CDKs 1, 2 and 4. Figure 2A–C (upper panel) shows the effect of 2,4,6-THBA on CDK1, 2 and 4 enzyme activity over a range of concentrations (62.5 µM to 1 mM for CDK1 and 2; and 125 µM to 1 mM for CDK4). The quantified data representing the degree of inhibition is shown in the lower panel. The IC_50_ of 2,4,6-THBA was 580 ± 57 µM for CDK1, 262 ± 29 µM for CDK2 and 403 ± 63 µM for CDK4. These experiments also revealed that 2,4,6-THBA was most effective against CDK2 as compared to CDKs 1 and 4. The middle panels of Figure 2A–C show that each of the lanes contain similar levels of the substrate. In a separate experiment, we also tested the inhibitory effect of 2,4,6-THBA on CDK6 activity; however, owing to the inconsistency in the obtained results, these data are not reported.

### 2.2. Comparison of the Effect of 2,4,6-THBA, Phloroglucinol and 3,4,5-THBA on CDK Enzyme Activity

To investigate the importance of the carboxyl (–COOH) and hydroxyl (–OH) functional groups and their positions within the aromatic ring, two structurally related compounds, phloroglucinol and 3,4,5-THBA (gallic acid), were tested for their effect on CDK activity and compared against 2,4,6-THBA at a concentration of 500 µM.

**Figure 3:**
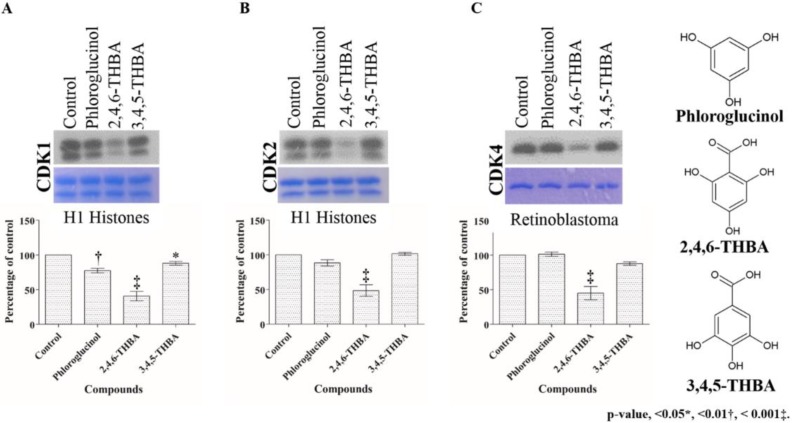
Comparison of the effect of phloroglucinol, 2,4,6-THBA and 3,4,5-THBA on CDK1 (**A**), CDK2 (**B**) and CDK4 (**C**) activity. Upper panel shows phosphorylation pattern of H1 histones (CDK1 and CDK2) and retinoblastoma protein (CDK4), in the presence of different compounds. The middle panel shows Coomassie Blue stained proteins. The lower panel shows quantification of the blots in the upper panel.

**Figure 4:**
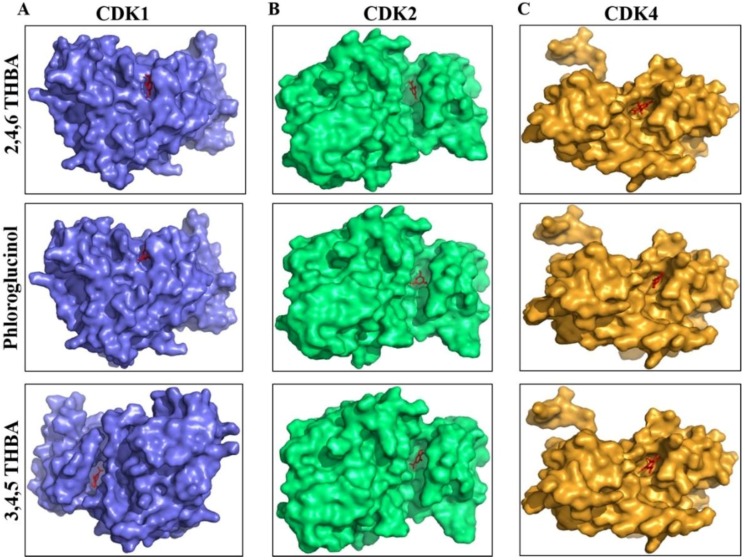
Space-filling model showing the orientation of the compounds and the potential binding pockets in CDK1 (**A**), CDK2 (**B**) and CDK4 (**C**) for 2,4,6-THBA (upper panel), phloroglucinol (middle panel) and 3,4,5-THBA (lower panel).

As shown in Figure 3, 2,4,6-THBA inhibited the activity of all three CDKs confirming the results obtained in Figure 2A–C. Interestingly, 3,4,5-THBA had a marginal effect, if any, on CDK1 and CDK4 and failed to inhibit CDK2, whereas phloroglucinol which lacks a –COOH group had no effect on CDK4 but slightly inhibited CDK1 and 2 (Figure 3, lower panel). These results suggest that the presence of a –COOH group and the relative positions of the –OH groups on the benzene ring are important for exhibiting effective CDK inhibition.

### 2.3. Molecular Docking Studies Show Potential Interactions of 2,4,6-THBA, Phloroglucinol and 3,4,5-THBA with CDKs

**Figure 5:**
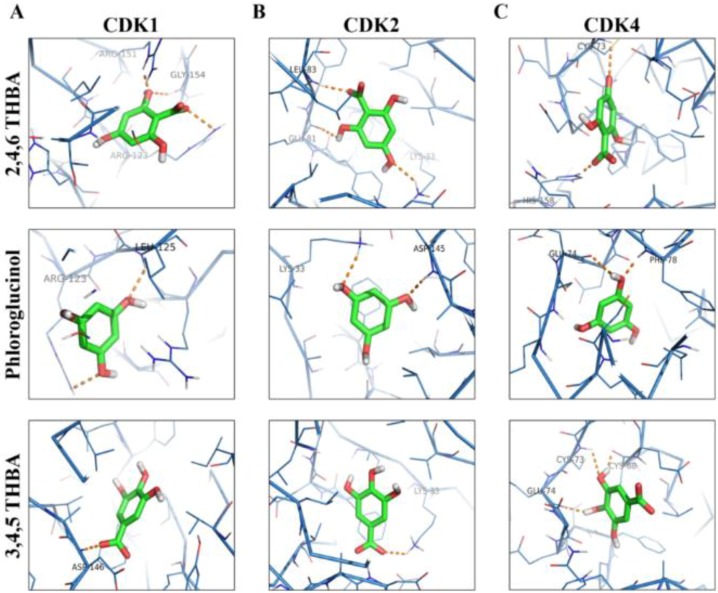
Molecular docking studies showing the potential interactions of 2,4,6-THBA (upper panel), phloroglucinol (middle panel) and 3,4,5-THBA (lower panel) with CDK1 (**A**), CDK2 (**B**) and CDK4 (**C**).

AutoDockVina was used to predict the potential interactions of 2,4,6-THBA, 3,4-DHBA, 4-HBA, phloroglucinol and 3,4,5-THBA with CDKs 1, 2 and 4. The binding free energy and hydrogen bond lengths were also determined. The results of the docking studies are shown in Table 1 and Figure 4 and Figure 5. The active site of the widely studied CDK2 enzyme has a total of 16 amino acids (Ile10, Gly11, Glu12, Val13, Val64, Glu81, Phe82, Leu83, Asp86, Asp127, Lys129, Gln131, Asn132, Leu134, Asp145, Thr165) [30]. All the compounds tested interacted with CDKs (except 4-HBA and CDK1) and utilized amino acids either residing in the active site or at alternate binding pockets. Docking studies revealed that 2,4,6-THBA potentially interacts with CDK1 through Arg123, Arg151, and Gly154; with CDK2 it utilizes Lys33, Gln81 and Leu83; and with CDK4, it interacts through Cys73 and His158. Phloroglucinol interacts with CDK1 using Leu125 and Arg123; with CDK2 it utilizes Asp145 and Lys33; and with CDK4 through Phe78 and Glu74. Interactions of 3,4,5-THBA with CDK1 occurs through Asp146; through Lys33 for CDK2; and with CDK4 utilizing Glu74, Cys73 and Cys68. Interactions of 2,4,6-THBA and phloroglucinol with CDK1 appear to be occurring in a pocket other than the active site; in contrast 3,4,5-THBA interacts with the amino acids in the active site. In CDK2 and CDK4, all three compounds seem to interact with amino acids in the active site. These results also suggest that 2,4,6-THBA can potentially interact with residues involved in CDK2-ATP-interaction.

### 2.4. 2,4,6-THBA Inhibits Cell Proliferation in Cell Lines Expressing Functional SLC5A8

Since 2,4,6-THBA effectively inhibited CDK activity in vitro, the possibility that it could inhibit cell proliferation of human CRC cells was explored. Surprisingly, when HCT-116 cells were exposed to 2,4,6-THBA (125–1000 μM) for 72 h, no decline in cell number was observed (Supplementary Figure S1). Additionally, the levels of cell cycle inhibitory proteins p21^Cip1^ and p27^Kip1^ also remained unaltered, and similar results were obtained in HT-29 and Caco-2 cell lines (data not shown). This prompted us to speculate that the unresponsiveness of these cells may be due to the lack of endogenous functional transporters for 2,4,6-THBA. We reasoned that 2,4,6-THBA being a monocarboxylic acid may be transported through the MCT SLC5A8 [31], which is expressed in the GI and proximal tubule for the transport of monocarboxylic acids [32,33]. Interestingly, in various cancers this protein is either inactivated through mutations (F251V) [33] or its expression is suppressed through promoter methylation [34]; this possibly explains the lack of an inhibitory effect of 2,4,6-THBA in CRC cell lines. Since a CRC cell line expressing a functional SLC5A8 was unavailable, we sought to determine the effect of 2,4,6-THBA on a breast cancer cell line (MDA-MB-231) previously characterized and reported to express this protein under the control of the tet-promoter (SLC5A8-pLVX) [35]. In contrast, parental MDA-MB-231 cells have been reported to express low levels of SLC5A8 [36].

**Figure 6:**
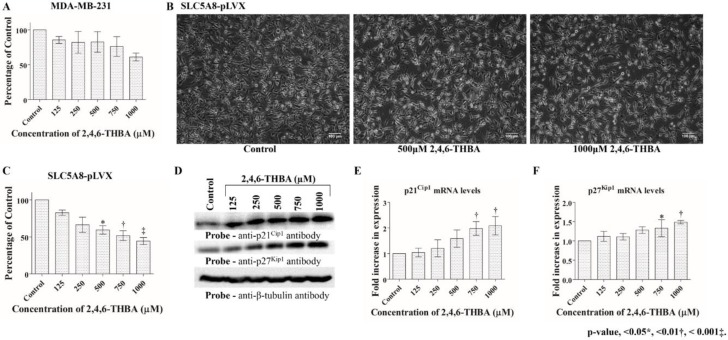
Effect of 2,4,6-THBA on MDA-MB-231 and SLC5A8-pLVX cells. (**A**) Graphic representation of the lack of an effect of 2,4,6-THBA on MDA-MB-231 cells. (**B**) Microscopy images showing the effect of 2,4,6-THBA in SLC5A8-pLVX cells. (**C**) Graphic representation showing the progressive decrease in cell number upon treatment with 2,4,6-THBA in SLC5A8-pLVX cells. (**D**) Western Blot analysis showing the increase in p21^Cip1^ and p27^Kip1^ protein levels in SLC5A8-pLVX cells in response to 2,4,6-THBA. (**E**,**F**) qPCR analysis showing fold-increase in p21^Cip1^ and p27^Kip1^ mRNA levels respectively in SLC5A8-pLVX cells in response to 2,4,6-THBA.

To investigate if the effect of 2,4,6-THBA required SLC5A8 transporter function, MDA-MB-231 and SLC5A8-pLVX cells were treated for 72 h at different concentrations from 125 µM to 1000 µM. Cells were counted for the inhibition of proliferation or analyzed for the induction of various cell cycle regulatory proteins namely p21^Cip1^, p27^Kip1^, CDK1, CDK2, CDK4, cyclin A2, cyclin B1 and cyclin D1. Figure 6A shows that no significant inhibition of cell proliferation was observed in MDA-MB-231 cells at concentrations from 125–750 μM; however, a reduction was observed at 1000 μM. Consistent with this, 2,4,6-THBA upregulated p21^Cip1^ and p27^Kip1^ protein levels at 1000 μM (data not shown). In contrast, in SLC5A8-pLVX cells, there was a clear decrease in cell density and number at all concentrations tested, indicative of an inhibitory effect on cell proliferation (Figure 6B,C). The decreased cell number correlated with increased levels of p21^Cip1^ and p27^Kip1^ (Figure 6D). In these lysates, protein levels of CDK1, CDK4, cyclin A2 and cyclin D1 remained unaltered while levels of CDK2 and cyclin B1 were reduced at higher concentrations (≥750 μM; Supplementary Figure S2). Further examination of mRNA levels revealed an increase of p21^Cip1^ at all concentrations whereas p27^Kip1^ levels were upregulated beginning at 500 µM (Figure 6E,F), suggesting that 2,4,6-THBA is an effective anti-proliferative agent. Collectively, these results indicate that SLC5A8 is a likely transporter for 2,4,6-THBA, and that its uptake contributes to the observed increase in the levels of p21^Cip1^ and p27^Kip1^, leading to inhibition of cell proliferation.

### 2.5. 2,4,6-THBA Is Taken Up by Cells Expressing Functional SLC5A8

**Figure 7:**
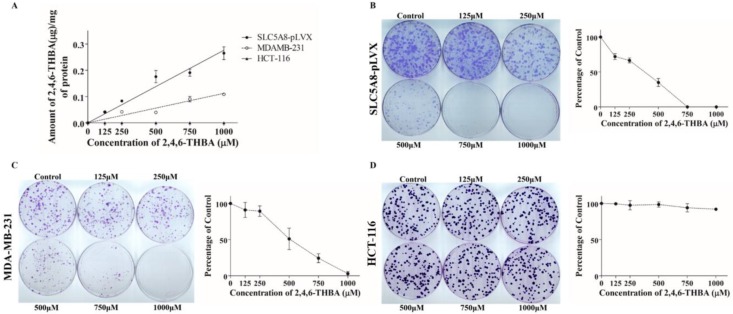
(**A**) HPLC analysis showing uptake of 2,4,6-THBA after 72 h in SLC5A8-pLVX, MDA-MB-231 and HCT-116 cells. (**B**–**D**) effect of 2,4,6-THBA (14–21 days) on colony formation in SLC5A8-pLVX, MDA-MB-231 and HCT-116 cells respectively. Quantification of the data is shown beside the crystal violet stained image of the colonies.

In independent experiments, using HPLC, cellular uptake of 2,4,6-THBA by SLC5A8-pLVX cells, MDA-MB-231 and HCT-116 cells was investigated to further confirm that SLC5A8 is a transporter for 2,4,6-THBA. Figure 7A demonstrates the dose-dependent uptake of 2,4,6-THBA by various cell-lines. In SLC5A8-pLVX cells, uptake was observed at 125 µM and increased linearly up to 1000 µM. As anticipated, reduced uptake was observed in the parental MDA-MB-231 cells with lower SLC5A8 expression (Supplementary Figure S3). Importantly, in HCT-116 cells, there was no uptake of 2,4,6-THBA despite the detection of SLC5A8 in the cell lysate (Supplementary Figure S3), which may be due to the presence of a dysfunctional transporter as previously reported [36]; this is also consistent with the lack of inhibition of proliferation in these cells (Supplementary Figure S1). Together, these results implicate SLC5A8 in the transport of 2,4,6-THBA.

### 2.6. 2,4,6-THBA Inhibits Colony Formation in Cells Expressing Functional SLC5A8

Clonogenic assay was performed to demonstrate the effectiveness of 2,4,6-THBA against colony formation in SLC5A8-pLVX, MDA-MB-231, HCT-116 and HT-29 cells. Figure 7B demonstrates that in SLC5A8-pLVX cells, 2,4,6-THBA dose dependently decreased the colony formation beginning at 125 µM with a 50% reduction observed around 400 µM. In addition to the decrease in the number of colonies, 2,4,6-THBA caused a reduction in the size of the colonies. In the MDA-MB-231 cells, 2,4,6-THBA also reduced the number of colonies after 250 μM with 50% reduction observed at about 446 μM (Figure 7C). In contrast, the colony formation in HCT-116 (Figure 7D) and HT-29 cells (Supplementary Figure S4A) remained unaffected at all concentrations. This further demonstrates that SLC5A8 is a transporter for 2,4,6-THBA and its uptake leads to inhibition of cell proliferation.

### 2.7. Cell Cycle Analysis and Apoptosis Assay

**Figure 8:**
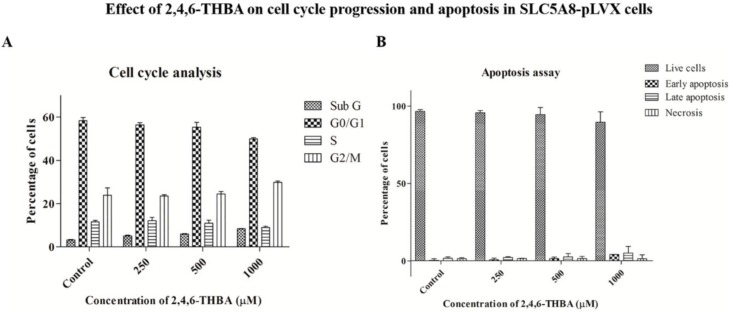
Graphic representation of (**A**) the cell cycle analysis and (**B**) apoptosis assay following treatment with 2,4,6-THBA in SLC5A8-pLVX cells. The percentage of cells in each stage was determined through flow cytometry.

We next determined the effect of 2,4,6-THBA on cell cycle arrest and apoptosis in SLC5A8-pLVX cells. Figure 8A demonstrates that no significant shift nor accumulation of a specific phase was observed in different stages of the cell cycle under treated conditions as compared to the untreated control. Interestingly, there was also no change in the number of cells undergoing apoptosis following 2,4,6-THBA treatment (Figure 8B). These results when combined with the cell count data obtained in Figure 6C suggests that 2,4,6-THBA is likely to slow down the rate of proliferation without causing an accumulation at any specific stage of the cell cycle, implying that the inhibition may be equal across all phases of the cell cycle. This is further supported by the earlier observation in Figure 2 that 2,4,6-THBA is an effective inhibitor of CDKs 1, 2 and 4. Lack of apoptosis observed also indicates that 2,4,6-THBA is likely to be a cytostatic agent rather than a cytotoxic agent at the concentrations tested.

### 2.8. Effect of Other Flavonoid Metabolites (4-HBA; 3,4-DHBA; and 3,4,5-THBA) on Clonal Formation

Next, the effect of metabolites generated from the flavonoid B-ring namely 4-HBA, 3,4-DHBA and 3,4,5-THBA on colony formation was investigated for comparison with 2,4,6-THBA. The data in Figure 3 showed that 3,4,5-THBA did not inhibit CDK activity, and our previous observations indicated that 3,4-DHBA is also incapable of inhibiting CDK1 [28]. Despite the lack of CDK inhibition by these compounds, the possibility that they may exhibit their known anti-proliferative activity [24,25,26,27] following uptake through SLC5A8 was considered. Consistent with previous reports in other cell lines, 3,4-DHBA and 3,4,5-THBA efficiently inhibited clonal formation in SLC5A8-pLVX, MDA-MB-231, and HCT-116 cells (Supplementary Figures S5 and S6). Inhibition of cell proliferation with 3,4-DHBA was observed beginning at a concentration of 15.62 μM for SLC5A8-pLVX, 7.3 μM for MDA-MB-231 and 125 μM for HCT-116 cells. Inhibition with 3,4,5-THBA was observed starting at 7.3 μM in all cell-lines. The lack of CDK-inhibition by 3,4-DHBA [28] and 3,4,5-THBA (Figure 3) and the results obtained in clonogenic assay clearly indicated that their mechanism of action is CDK-independent. Interestingly, 4-HBA failed to inhibit clonal formation at all concentrations tested (Supplementary Figures S4D and S7) which suggests that the flavonoid metabolites are highly selective in their actions.

The ability of 3,4-DHBA and 3,4,5-THBA to inhibit proliferation in HCT-116 cells (Supplementary Figures S5 and S6) was surprising as in these cells the structurally related 2,4,6-THBA failed to inhibit cell proliferation. These contrasting results prompted the study of their uptake in SLC5A8-pLVX, MDA-MB-231 and HCT-116 cells. The data obtained from HPLC analysis showed that 3,4-DHBA and 3,4,5-THBA were not taken up whereas cellular uptake of 4-HBA was consistently observed in all three cell lines tested (Supplementary Figure S8). Inhibition of cell proliferation by 3,4-DHBA and 3,4,5-THBA in all three cell lines without their cellular uptake suggests that the biological targets for these compounds are extracellular. The uptake of 4-HBA in all cell lines might be mediated either through an alternative transporter or it passively diffuses across the cell membrane.

## 3. Discussion

In this research paper, we report several novel observations including a mechanism by which flavonoid metabolites known to be generated in the GI tract may exert their anti-cancer effects against CRC through CDK-dependent and -independent pathways. This study investigated 2,4,6-THBA generated from flavonoid A-ring and 4-HBA, 3,4-DHBA and 3,4,5-THBA from the flavonoid B-ring for their potential to inhibit cancer cell growth. Our results demonstrated that the inhibitory effect of 2,4,6-THBA on purified CDKs in vitro was dose dependent with IC_50_ observed between 262–580 µM, while molecular docking studies revealed the key amino acid interactions. We also identified SLC5A8 as a transporter for 2,4,6-THBA, and using SLC5A8-pLVX cells, we demonstrated that its cellular uptake for 72 h was sufficient to cause induction of p21^Cip1^ and p27^Kip1^, leading to inhibition of cell proliferation. Although endogenous SLC5A8 was detected in HCT-116 cells, they were insensitive to 2,4,6-THBA treatment. Additionally, using clonogenic assays, we showed that 2,4,6-THBA was highly effective in inhibiting clonal formation in the cell lines expressing functional SLC5A8. The IC_50_ of clonal inhibition corresponded to the IC_50_ of CDK inhibition, suggesting that 2,4,6-THBA may mediate its effect through a CDK-dependent pathway. We believe the inhibition of proliferation can occur through at least two different mechanisms—one by direct binding of 2,4,6-THBA to CDKs leading to their inhibition and the other through upregulation of CDK inhibitory proteins p21^Cip1^ and p27^Kip1^. Although 3,4-DHBA and 3,4,5-THBA were incapable of inhibiting CDKs, they efficiently inhibited cell proliferation in all cell lines tested including HCT-116 and HT-29 (Supplementary Figures S4–S6). This suggests that the mechanism of inhibition by 3,4-DHBA and 3,4,5-THBA is both an SLC5A8- and CDK-independent phenomenon. Not all metabolites exhibited anti-proliferative effects as 4-HBA although taken up by all cell lines tested was ineffective in inhibiting cell proliferation which suggests that the metabolites are selective in their actions. Our results, for the first time demonstrate a role for flavonoid metabolites in the inhibition of cancer cell growth occurring through both CDK-dependent and -independent mechanisms, and also establishes a functional role for SLC5A8 in the transport of 2,4,6-THBA.

Molecular docking studies indicated that 2,4,6-THBA binds to CDKs 1, 2 and 4 at specific sites within these enzymes. The CDK structure is highly conserved among the different family members with a bi-lobe fold that harbors a conserved ATP-binding domain [37,38]. The interactions of 2,4,6-THBA with CDKs 2 and 4 at the ATP-binding site may explain the enhanced inhibition of enzyme activity in comparison to the lesser inhibition of CDK1 by 2,4,6-THBA which interacts at an allosteric site. Interestingly, the structurally related compound 3,4,5-THBA failed to inhibit CDK activity although docking studies provided evidence of its binding to all CDKs (Table 1 and Figure 5). This suggests that the presence of hydroxyl groups at specific positions on the benzene ring in these compounds confers the ability to bind and cause enzyme inhibition. The –COOH group seems to be important in increasing the potency of this inhibition as phloroglucinol which lacks the –COOH group was only marginally effective in inhibiting CDK1 and CDK2. Therefore, we suggest that both the presence of –COOH and the position of the –OH groups are important for efficient CDK inhibition. Further studies involving kinetics of inhibition are required to fully understand the nature of these interactions and its implications towards inhibition of enzyme activity.

Detection of 2,4,6-THBA in SLC5A8-pLVX cells through HPLC suggests that SLC5A8 is a natural transporter for this monocarboxylic acid. The attributed physiological functions of SLC5A8 includes transport of short chain fatty acids (SCFAs), salicylates, propionate, butyrate, lactate, pyruvate, and benzoates to name a few [33]. Numerous studies have shown that SLC5A8 is likely to be a tumor suppressor protein [34,35] and the mechanism is predicted to involve inhibition of histone deacetylases (HDACs) through the uptake of SCFAs by these transporters [33,36]. It is a sodium-coupled MCT and is expressed in the apical membrane of the colonic and intestinal epithelium [32]. It is silenced in many cancers including colon, thyroid, kidney, stomach, brain, breast, pancreas and prostate. Studies suggest that silencing of SLC5A8 contributes to tumorigenesis [39]. We speculated that the lack of response observed following 2,4,6-THBA treatment of HCT-116 and other CRC cell lines is due to the absence of a wild-type functional SLC5A8. In support of this view, no cellular uptake of 2,4,6-THBA was observed in these cells. Importantly, in SLC5A8-pLVX cells expressing the functional SLC5A8, 2,4,6-THBA not only induced p21^Cip1^ and p27^Kip1^ RNA and protein expression, but also reduced cell proliferation. Our demonstration that 2,4,6-THBA inhibits cell growth in cancer cells expressing functional SLC5A8 suggests that such a phenomenon may indeed exist in vivo and may contribute to its anti-cancer effects against CRC.

The mechanism by which 2,4,6-THBA inhibits CDK activity requires additional investigations. Unlike the conventional drugs Palbociclib and Ribociclib that target CDK4/6 and cause G0/G1 cell cycle arrest [40], 2,4,6-THBA appears to target CDKs 1, 2, and 4. The lack of inhibition at any particular stage of the cell-cycle observed in our study (Figure 8A) may be attributed to the effect of 2,4,6-THBA on multiple CDKs (Figure 2A–C), leading to a decrease in the overall growth rate without causing accumulation of cells at any given stage. Exposure of SLC5A8-pLVX cells to 2,4,6-THBA at concentrations from 125–1000µM slowed the proliferation (Figure 6C) without causing significant apoptosis (Figure 8B) suggesting that it is more likely to be a cytostatic rather than a cytotoxic agent.

The exposure of SLC5A8-pLVX cells as well as MDA-MB-231 cells to 2,4,6-THBA for 14–21 days resulted in a decrease in colony formation. The ability of 2,4,6-THBA to inhibit cell proliferation in MDA-MB-231 cells was surprising considering the low expression levels of SLC5A8 and the fact that acute treatment (72 h) with 2,4,6-THBA did not cause any significant decrease in cell proliferation. This discrepancy between acute and chronic treatment in MDA-MB-231 cells can be explained by the limited uptake of 2,4,6-THBA during acute exposure, whereas chronic exposure of these cells to 2,4,6-THBA could result in significant accumulation sufficient to cause inhibition of cell proliferation. This was consistent with the HPLC data that showed lower uptake of 2,4,6-THBA in MDA-MB-231 cells when compared to SLC5A8-pLVX cells (Figure 7A). Consistent with the previously reported lack of functional SLC5A8 in HCT-116 cells [36], 2,4,6-THBA had no effect on the colony formation (Figure 7D), and HPLC also showed no uptake of 2,4,6-THBA in these cells (Figure 7A). These results suggest that the transporter activity of SLC5A8 is required for 2,4,6-THBA-mediated inhibition of cell proliferation.

Tumor progression involves distinct stages—initiation, promotion, progression and metastasis. Of these stages, it is suggested that chemo-preventive agents preferentially act within the initiation and promotion stages to reverse the process of carcinogenesis [41]. They are further classified into blocking agents and suppressing agents depending on their ability to prevent carcinogenesis and suppress neoplastic cell growth respectively [42]. Based on the properties reported for phenolic compounds [43,44] and the results presented in this study, we propose that 2,4,6-THBA can act as both—a blocking and a suppressing agent. Since it is a phenolic compound with multiple hydroxyl groups, it can act as a blocking agent and exert its effect through its anti-oxidant properties [45]. It can also be viewed as a suppressing agent through its direct inhibitory effect on CDKs 1, 2 and 4 leading to reduced rate of cell proliferation. Its anti-oxidant and anti-proliferative actions could collectively contribute to cancer prevention by slowing down the rate of cell proliferation and providing an opportunity to repair the DNA damage or for immune surveillance by Natural Killer- and cytotoxic T-cells for cancer cell destruction [46,47].

Although 3,4-DHBA and 3,4,5-THBA failed to inhibit CDK activity in vitro, their ability to inhibit colony formation in all cell lines tested irrespective of the SLC5A8 expression is also an important observation. It suggests that these compounds exert their anti-proliferative effect through an SLC5A8- and CDK-independent mechanism. These flavonoid metabolites have previously been shown to inhibit cancer cell growth through multiple signaling pathways [24,27]; however, no direct primary target(s) have been identified. It appears that depending upon the cell line tested, 3,4-DHBA and 3,4,5-THBA can inhibit colony formation within a range of 7.3–250 μM and 7.3–31.25 μM respectively. These concentrations are significantly lower compared to those of 2,4,6-THBA. The lack of detection of these compounds in HPLC studies suggests that they are not taken up by the cells, and hence we suggest that their primary target is likely to be extracellular.

**Figure 9:**
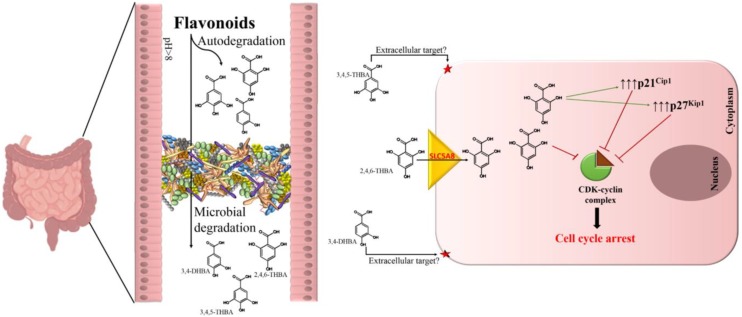
A model depicting how flavonoid metabolites may exert its chemo-preventive effects on colonic tissue to protect against CRC. We suggest that 2,4,6-THBA generated by auto-degradation of flavonoids or those through the action of microflora is taken up by the colonic epithelial cells through SLC5A8. Since SLC5A8 is expressed only in the apical surface (and not in the basolateral surface) [35], 2,4,6-THBA may accumulate to pharmacologically relevant concentrations within cells, sufficient to inhibit CDKs. This would lead to a reduced rate of cell proliferation through 2 distinct mechanisms—one by direct inhibition of CDKs by 2,4,6-THBA and the other through the upregulation of p21^Cip1^ and p27^Kip1^, possibly through an indirect pathway. The reduced rate of proliferation will provide an opportunity for DNA repair to occur or destruction of cancer cells through immune surveillance. The metabolites such as 3,4-DHBA and 3,4,5-THBA that are generated from flavonoids may also contribute to chemo-prevention by targeting extracellular proteins.

The discovery of the flavonoid metabolite 2,4,6-THBA as an inhibitor of CDKs and the demonstration of its ability to inhibit cell proliferation is a significant finding. Although the IC_50_ of 2,4,6-THBA and other compounds tested in this study are in micromolar concentrations, it could be argued that it is physiologically relevant in view of their abundance in the diet [8,9]. CDKs and other potential cellular targets probably have evolved to be less sensitive to these compounds to avoid complete inhibition or perturbance of cell cycle at lower concentrations. We suggest that the reported chemo-preventive actions of flavonoids could be explained at least in part through the ability of its metabolite 2,4,6-THBA to target CDKs by direct inhibition and through upregulation of CDK inhibitory proteins; this would tip the balance strongly towards cell-cycle arrest, leading to reduced cell proliferation. A model depicting how flavonoid metabolites may prevent the occurrences of CRC is shown in Figure 9. Since the metabolites are also generated through microbial degradation, it highlights the importance of GI microflora in the prevention of CRC.

Effective cancer prevention may therefore require both the chemo-preventive agents and the responsible partners for their degradation. Identification of the microbial species contributing to this process as well as the mechanism of action of the metabolites on GI tissue is an important area for future study. Additionally, in vivo studies would be required to further confirm the chemo-preventive role of 2,4,6-THBA against CRC.

## 4. Materials and Methods

### 4.1. Cell Lines

HCT-116, HT-29, Caco-2 and MDA-MB-231 cell lines were purchased from American Type Culture Collection (ATCC, Manassas, VA, USA). MDA-MB-231 cells expressing functional SLC5A8 (SLC5A8-pLVX cell line) was kindly provided by Dr. Puttur Prasad, Medical College of Georgia. The cells were cultured in their respective medium (RPMI media for both the MDA-MB-231 cells and McCoy’s 5A media for all other cell lines) containing 10% Fetal Bovine Serum (FBS) with antibiotics for 24 h before treatment with specified compounds for indicated times. SLC5A8-pLVX cells were grown in the presence of doxycycline (5 μg/mL) for the induction of SLC5A8. Authentication of cell lines was done by ATCC through their DNA-STR profile.

### 4.2. Reagents

2,4,6-THBA, 3,4-DHBA, 3,4,5-THBA, 4-HBA and trypsin-EDTA were obtained from Sigma Aldrich (St. Louis, MO, USA); H1 Histones and Immobilon membranes from EMD Millipore (Billerica, MA, USA); ^32^P γ-ATP from MP Biochemicals (Solon, OH, USA); RT-PCR reagents from New England Biolabs (NEB, Ipswich, MA, USA); qPCR reagents from Applied Biosystems (Foster City, CA, USA); Annexin V/7-AAD kit from Beckman Coulter (Miami, FL, USA); Super Signal™ West Pico Chemiluminescent Substrate, protease inhibitor tablets and all other chemicals were obtained from Thermo Fisher Scientific, Inc. (Waltham, MA, USA).

### 4.3. Recombinant Proteins and Antibodies

anti-p21^Cip1^, anti-p27^Kip1^, anti-CDK1, anti-CDK2, anti-CDK4, anti-cyclinA2, anti-cyclinB1, anti-cyclinD1, and anti-β tubulin antibodies were purchased from Cell Signaling Technology (Danvers, MA, USA); anti-SLC5A8 from Invitrogen (Carlsbad, CA, USA); goat anti-rabbit and goat anti-mouse antibodies were obtained from Bio-Rad (Hercules, CA, USA). CDK1/Cyclin B1, CDK2/Cyclin A2, CDK4/Cyclin D1, Retinoblastoma (C-term) and kinase buffer were purchased from SignalChem (Richmond, BC, Canada).

### 4.4. Cell Lysate Preparation and Western Blotting

Following treatment with compounds at the specified concentrations, cells were washed with 1X phosphate buffered saline (PBS) and lysates were prepared as previously described [48]. Fifty micrograms of total protein was separated on an 8 or 12% polyacrylamide gel, transferred to immobilon membrane and immunoblotted with indicated antibodies.

### 4.5. In Vitro CDK Assay

In vitro CDK assays were performed as described by the manufacturer. For this, purified enzyme was aliquoted into the reaction buffer and incubated with indicated compounds at various concentrations for 10 min at room temperature. The reaction mixture was incubated with kinase buffer containing 15 µM ATP, 2 µCi of [γ ^32^P] ATP, 5 µg of H1 Histone or retinoblastoma, at 30 °C for 20 min in a final volume of 50 μL. The reactions were halted by adding EDTA to a final concentration of 20 mM and addition of 4× loading buffer. The samples were boiled for 10 min, analyzed by 8 or 10% SDS-PAGE, stained using coomassie brilliant blue (R250), dried and exposed to X-ray film. NIH ImageJ software was used to quantify the intensities of the bands.

### 4.6. Molecular Docking Studies

The crystallographic three dimensional structures of CDK1 (4Y72 A chain), CDK2 (1FIN A chain) and CDK4 (3G33 A chain) were retrieved from the Protein Data Bank (PDB). Energy minimization for these proteins was performed using Gromacs 3.3.1 package utilizing GROMOS96 force field [49]. The energy-minimized molecules were used as the receptors for virtual small molecule docking with 2,4,6-THBA, 3,4-DHBA, 3,4,5-THBA, 4-HBA and phloroglucinol using AutoDockVina. The results were visualized by PYMOL molecular graphics system version 1.3.

### 4.7. HPLC Analysis

High Performance Liquid Chromatography (HPLC) was utilized to determine the uptake of flavonoid metabolites. Cells were exposed to different compounds at indicated concentrations and lysates were prepared as described previously [28]. Protein concentration was determined; 300 μL of the lysates were taken and proteins were precipitated using 15 μL of trifluoracetic acid (TFA). Samples were centrifuged at 14,000 rpm for 5 min, the supernatant was transferred to fresh tubes and the pH was adjusted to about 4–5. Finally, 300 μL of acidified methanol was added to the samples. HPLC analysis was conducted using a Waters HPLC system (Milford, MA, USA) equipped with a 1525 binary pump and a W2998 PDA detector. An isocratic method was used to elute the compound in reverse phase using a ZORBAX SB-CN, 5 µm, 4.6 × 250 mm column (Agilent, Santa Clara, CA, USA). The mobile phase contained 20 mM ammonium acetate (adjusted to pH 4.0 with acetic acid) and methanol (90:10 ratio), with a flow rate of 0.7 mL/min. The injection volume was 40 μL. Compounds were detected at a wavelength of 260 nm. Quantification was done by generating a standard graph using known amounts of each compound.

### 4.8. RNA Isolation and qRT-PCR

Total RNA was isolated from treated and untreated cells as previously described using TRIzol reagent [50]. RT-PCR was performed according to the manufacturer’s instructions (NEB). Briefly, total RNA from 2,4,6-THBA treated cells were isolated and reverse transcribed for 1 h at 42 °C using M-MuLV reverse transcriptase followed by amplification of the cDNA product. The qPCR experiment was set by adding equal volumes of each template to the SYBR Fast Master mix along with ROX dye (Applied Biosystems) and 5 pmols of each primer (p21^Cip1^ Forward: AGCTCAATGGACTGGAAGG Reverse: TGGATGAGGAAGGTCGCT; p27^Kip1^ Forward: GCTGAG GAACTGACGTGG Reverse: AGGGCAGTGAGGATAGGT). The final volume was adjusted to 20 µL using nuclease free water (NEB) and the reaction mixture was amplified through one cycle of 50 °C for 2 min, 95 °C for 2 min; 40 cycles of 95 °C for 15 s, 60 °C for 1 min and 72 °C for 1 min; and one melt cycle of 95 °C for 15 s, 60 °C for 1 min and 95 °C for 15 s. GAPDH was used as internal control for the qPCR reaction (Forward: CCACTCCTCCACCTTTGAC Reverse: ACCCTGTTGCTGT AGCCA).

### 4.9. Flow Cytometric Analysis

SLC5A8-pLVX cells were treated for 72 h with varying concentrations of 2,4,6-THBA. Adherent cells were collected by trypsinization and pooled with floating cells, washed with 1X PBS. Cell cycle analysis was performed by adding Vybrant^®^ DyeCycle™ Green Stain (Invitrogen, Carlsbad, CA, USA) at a final concentration of 250 nM to 1 mL cell suspension. The samples were analyzed by flow cytometry following incubation for 30 min at 37 °C. To detect apoptosis, cells were stained with an Annexin V/7-AAD kit as previously described [7]. All experiments were carried out by the CytoFLEX flow cytometer (Beckman Coulter, Miami, Indianapolis, IN, USA) using CytExpert 2.0 software (Beckman Coulter, Indianapolis, IN, USA).

### 4.10. Clonogenic Assay

Clonogenic assays were performed as previously described [51]. Cells were seeded at a density of 500 cells/100 mm plate and grown for 48 h following which specified compounds were added at the concentrations indicated. The spent media was replaced with fresh media containing the respective compounds every 5–6 days. Cells were incubated for 14–21 days, fixed with 100% methanol for 20 min, and stained with 0.5% crystal violet prepared in 25% methanol. The colonies were then photographed and quantified using the ImageJ software (NIH, Bethesda, MD, USA).

### 4.11. Statistical Analysis

All experiments were repeated 3–6 times independently of each other. One-way ANOVA followed by Tukey’s post-hoc analysis was used to analyze group differences to the control, and significance was defined at *p* < 0.05.

## 5. Conclusions

In this research paper, using a variety of biochemical, molecular biology and computational approaches, we report that the flavonoid metabolite 2,4,6-trihydroxybenzoic acid (2,4,6-THBA) inhibits CDK enzyme activity and exhibits potent anti-proliferative effects. We showed that cellular uptake of 2,4,6-THBA required the expression of SLC5A8, a monocarboxylic acid transporter. Investigations were also carried out to determine the effectiveness of three other metabolites—4-hydroxybenzoic acid (4-HBA), 3,4-dihydroxybenzoic acid (3,4-DHBA) and 3,4,5-THBA. Of these only 3,4-DHBA and 3,4,5-THBA inhibited cancer cell proliferation and this was independent of both SLC5A8 transport and CDK inhibition. These findings for the first time, suggests that the flavonoid metabolite 2,4,6-THBA along with 3,4-DHBA and 3,4,5-THBA may contribute to the chemo-preventive effects of flavonoids against CRC. In addition, our studies also highlight the need of further investigations directed towards the role(s) played by flavonoid metabolites in the prevention of cancer. Our finding that 2,4,6-THBA is an effective inhibitor of CDKs that acts as an anti-proliferative agent suggests that it has the potential to be developed into a novel class of CDK inhibitors.

## Figures and Tables

**Table 1:** Free energy binding values and hydrogen bond lengths for the interaction of 2,4,6-THBA, phloroglucinol, 3,4-DHBA, 4-HBA and 3,4,5-THBA with CDK1, CDK2 and CDK4.

S.No	Receptor	Ligand	Interacting Amino Acids	Measurement (A°)	No of H Bonds	Energy Value (kCal/mol)
1.	CDK1	2,4,6-THBA	Arg123, Gly154, Arg151	2.5, 2.4, 2.0	3	−6.0
		3,4,5-THBA	Asp146	2.1	1	−6.1
		Phloroglucinol	Leu125, Arg123	2.4, 2.6	2	−4.9
		3,4-DHBA	Asp146	2.8	1	−6.3
		4-HBA	-	-	0	−6.4
2	CDK2	2,4,6-THBA	Leu83, Gln81, Lys33	2.2, 2.3, 2.4	3	−5.6
		3,4,5-THBA	Lys33	2.5	1	−5.8
		Phloroglucinol	Asp145, Lys33	2.2,2.4	2	−5.2
		3,4-DHBA	Leu83(2), Glu81	3.2, 3.1, 2.0	3	−5.8
		4-HBA	Lys33	2.9	1	−5.7
3.	CDK4	2,4,6-THBA	His158, Cys73	21, 2.8	2	−5.8
		3,4,5-THBA	Glu74, Cys73, Cys68	2.2, 2.8, 1.9	3	−5.8
		Phloroglucinol	Phe78, Glu74	2.1, 2.7	2	−5.0
		3,4-DHBA	Cys68, Glu75	2.5, 2.7	2	−5.7
		4-HBA	His158	3.2	1	−5.7

## Supplementary Materials

The following are available online at https://www.mdpi.com/2072-6694/11/3/427/s1, Figure S1: Effect of 2,4,6-THBA on HCT-116 cell proliferation; Figure S2: Western Blot analysis showing the levels of various CDKs and cyclins in SLC5A8-pLVX cells in response to 2,4,6-THBA; Figure S3: Western Blot demonstrating the expression levels of SLC5A8 protein in HCT-116, HT-29, MDA-MB-231 and SLC5A8-pLVX cell lines; Figure S4: Effect of 2,4,6-THBA (A), 3,4,5-THBA (B), 3,4-DHBA (C) and 4-HBA (D) on colony formation in HT-29 cells; Figure S5: Effect of different concentrations of 3,4-DHBA on colony formation in SLC5A8-PLVX (A), MDA-MB-231 (B), and HCT-116 (C) cells; Figure S6: Effect of different concentrations of 3,4,5-THBA on colony formation in SLC5A8-PLVX (A), MDA-MB-231 (B), and HCT-116 (C) cells; Figure S7: Effect of different concentrations of 4-HBA on colony formation in SLC5A8-PLVX (A), MDA-MB-231 (B), and HCT-116 (C) cells; Figure S8: HPLC analysis showing the cellular uptake in SLC5A8-pLVX, MDA-MB-231 and HCT-116 cells following incubation with 3,4-DHBA (A), 3,4,5-THBA (B) and 4-HBA (C) in the cytosol.
