# Supplementary material for: The Flavonoid Metabolite 2,4,6-Trihydroxybenzoic Acid Is a CDK Inhibitor and an Anti-Proliferative Agent: A Potential Role in Cancer Prevention
Source: Cancers (Basel). 2019 Mar 26;11(3):427. doi: 10.3390/cancers11030427 (PMC6468648; doi:10.3390/cancers11030427)
Supplement: Supplementary file 1 [file cancers-11-00427-s001.pdf]

cancers

The Flavonoid Metabolite 2,4,6-Trihydroxybenzoic Acid Is a CDK Inhibitor and an Anti-Proliferative Agent: A Potential Role in Cancer Prevention

Ranjini Sankaranarayanan, Chaitanya K. Valiveti, D. Ramesh Kumar, Severine Van slambrouck, Siddharth S. Kesharwani, Teresa Seefeldt, Joy Scaria, Hemachand Tummala and G. Jayarama Bhat

Supplementary Material

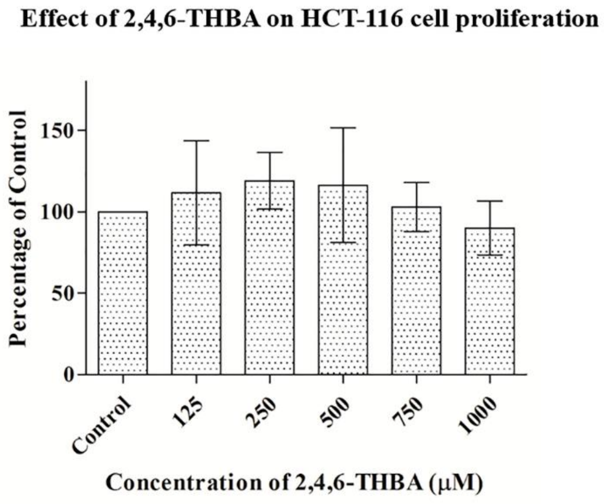

Figure S1. Effect of 2,4,6-THBA on HCT-116 cell proliferation. Cells were treated with 2,4,6-THBA for 72 h. Floating cells were collected, the adherent cells were washed, trypsinized and pooled with the floating cells and counted.

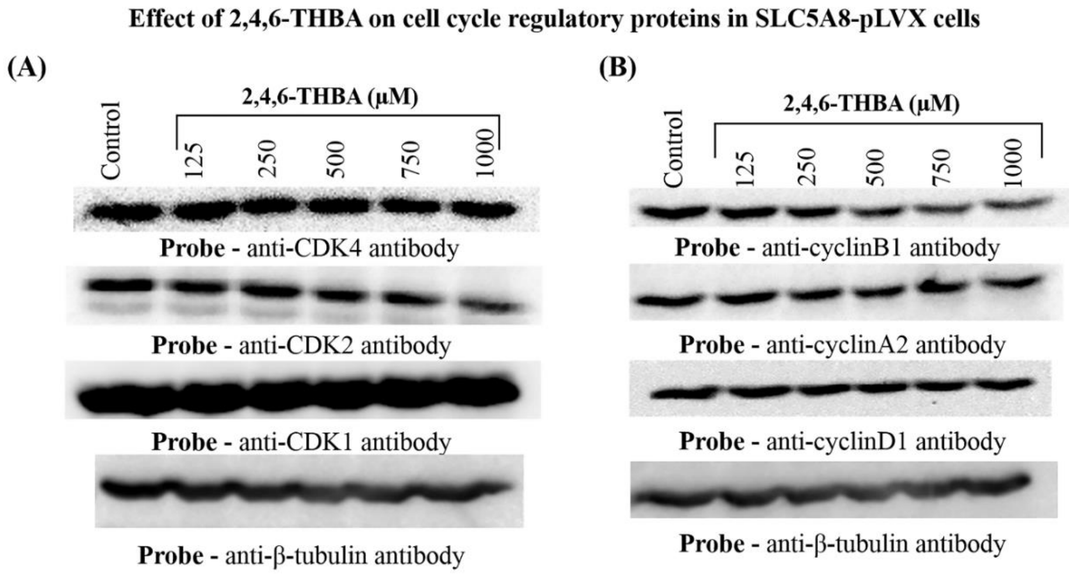

Figure S2. Western Blot analysis showing the levels of various CDKs and cyclins in SLC5A8-pLVX cells in response to 2,4,6-THBA.

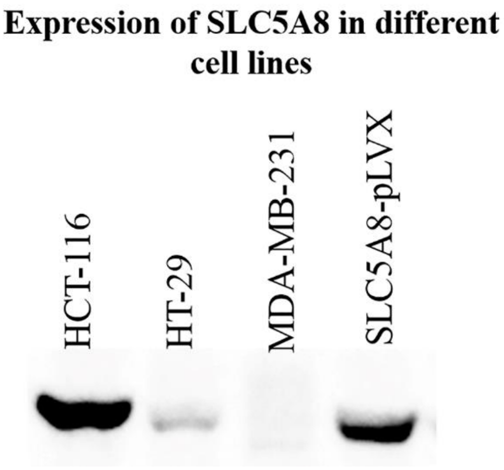

Figure S3. Western Blot demonstrating the expression levels of SLC5A8 protein in HCT-116, HT-29, MDA-MB-231 and SLC5A8-pLVX cell lines. Despite the expression of SLC5A8 in HCT-116 cells, uptake of 2,4,6-THBA was not observed (Figure 6A). In contrast, uptake was observed in SLC5A8-pLVX cells expressing the functional transporter. Consistent with the low expression of SLC5A8 in MDA-MB-231cells, low levels of 2,4,6-THBA was observed in these cells.

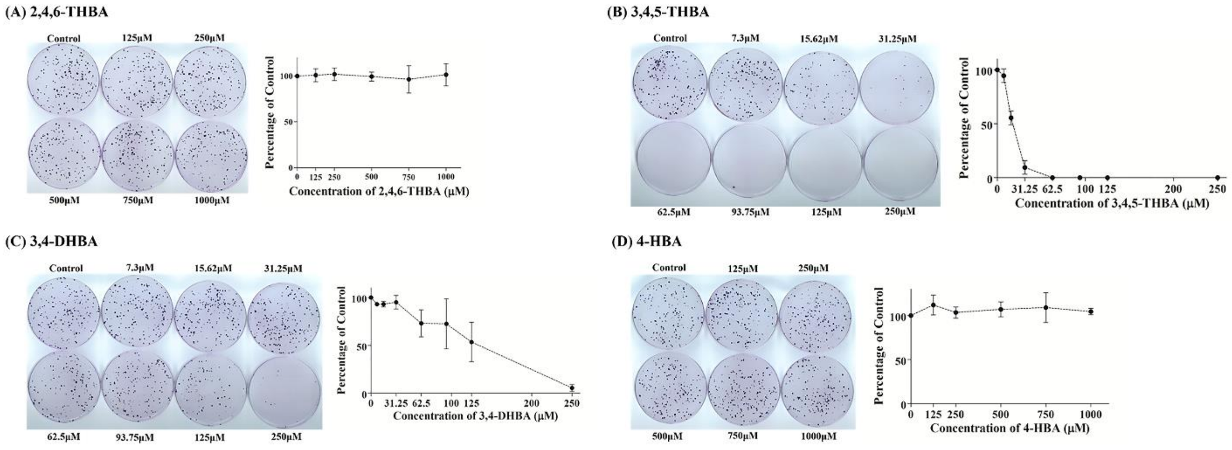

Figure S4. Effect of 2,4,6-THBA (**A**), 3,4,5-THBA (**B**), 3,4-DHBA (**C**) and 4-HBA (**D**) on colony formation in HT-29 cells.

Colony formation assay following treatment with 3,4-DHBA

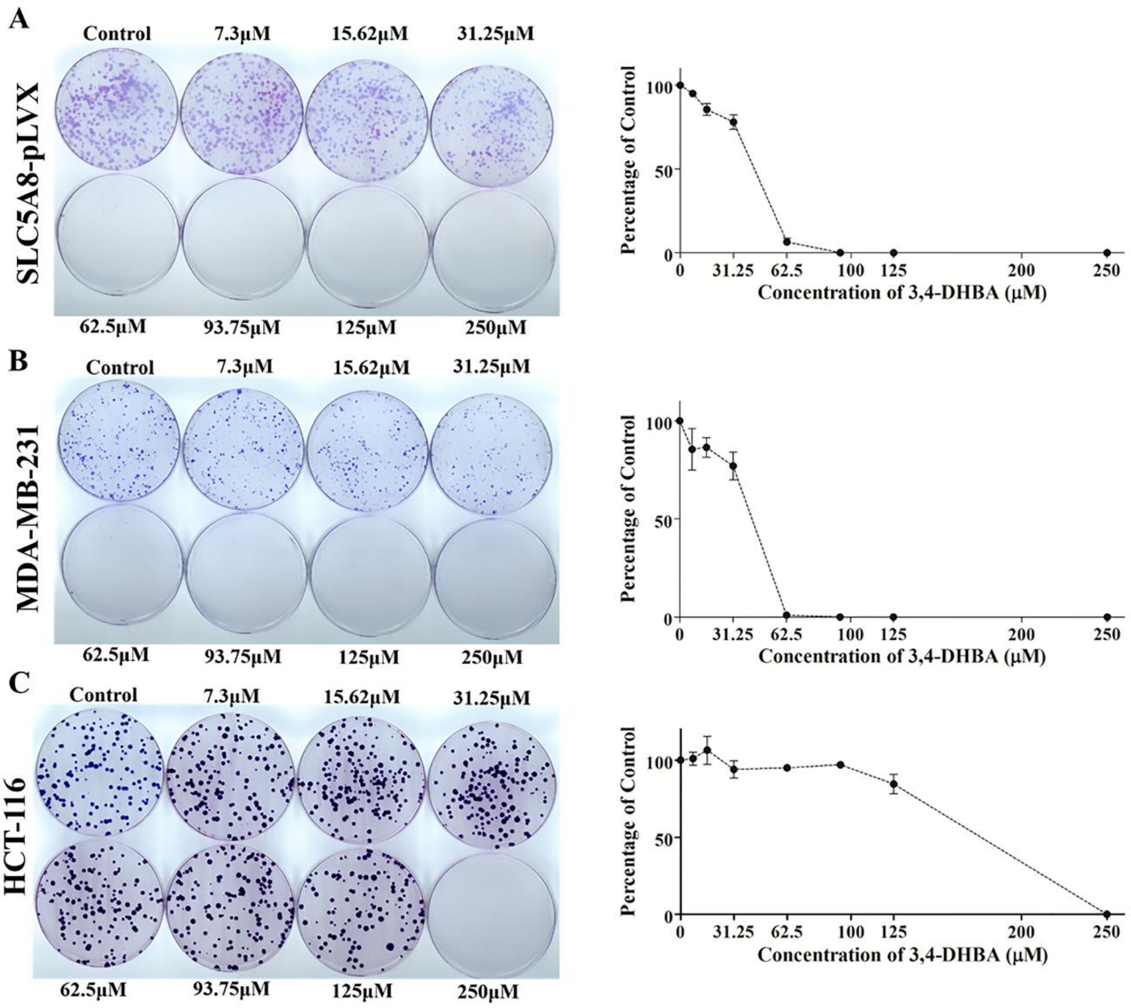

Figure S5. Effect of different concentrations of 3,4-DHBA on colony formation in SLC5A8-PLVX (**A**), MDA-MB-231 (**B**), and HCT-116 (**C**) cells.

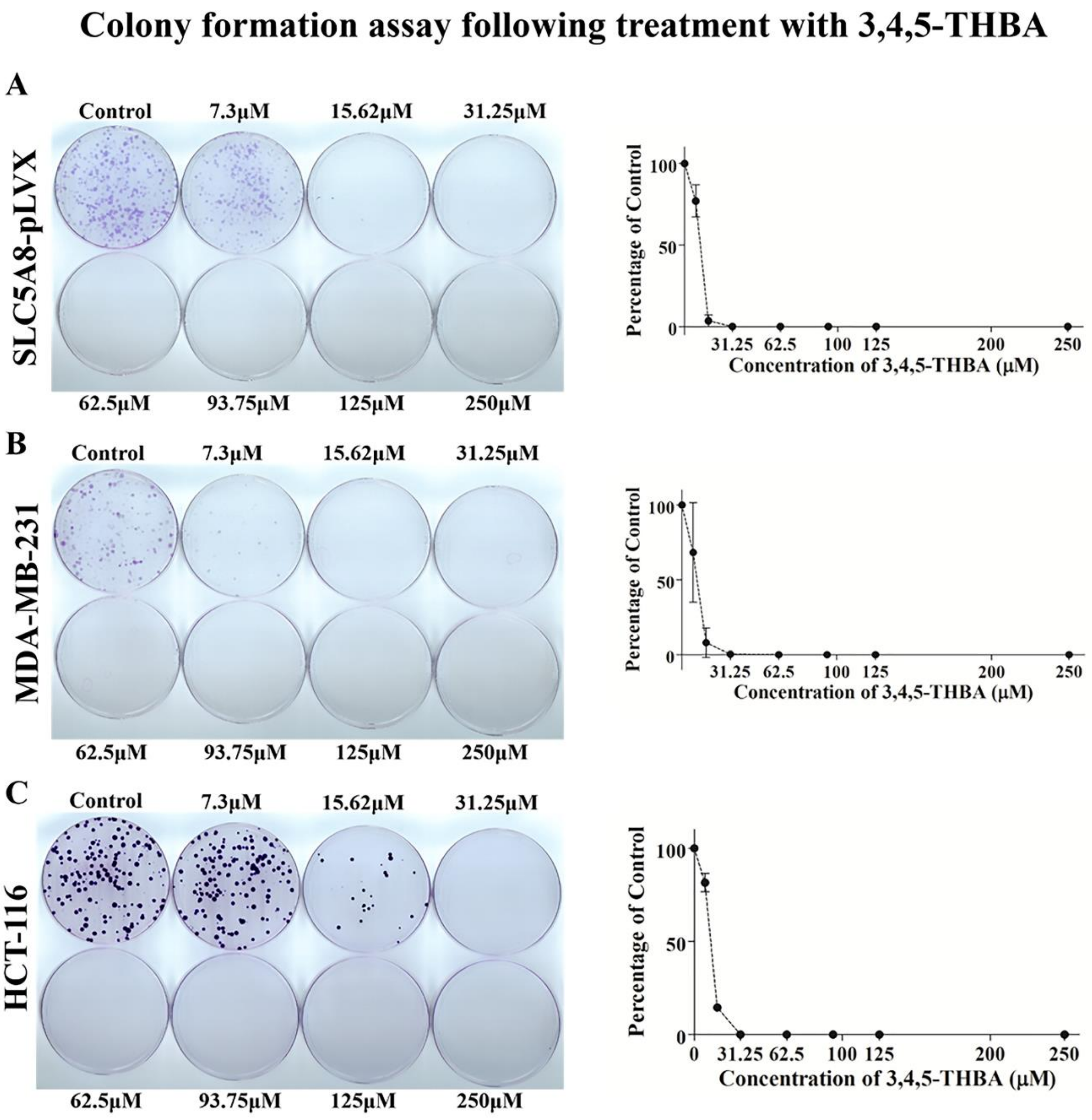

Figure S6. Effect of different concentrations of 3,4,5-THBA on colony formation in SLC5A8-PLVX (**A**), MDA-MB-231 (**B**), and HCT-116 (**C**) cells.

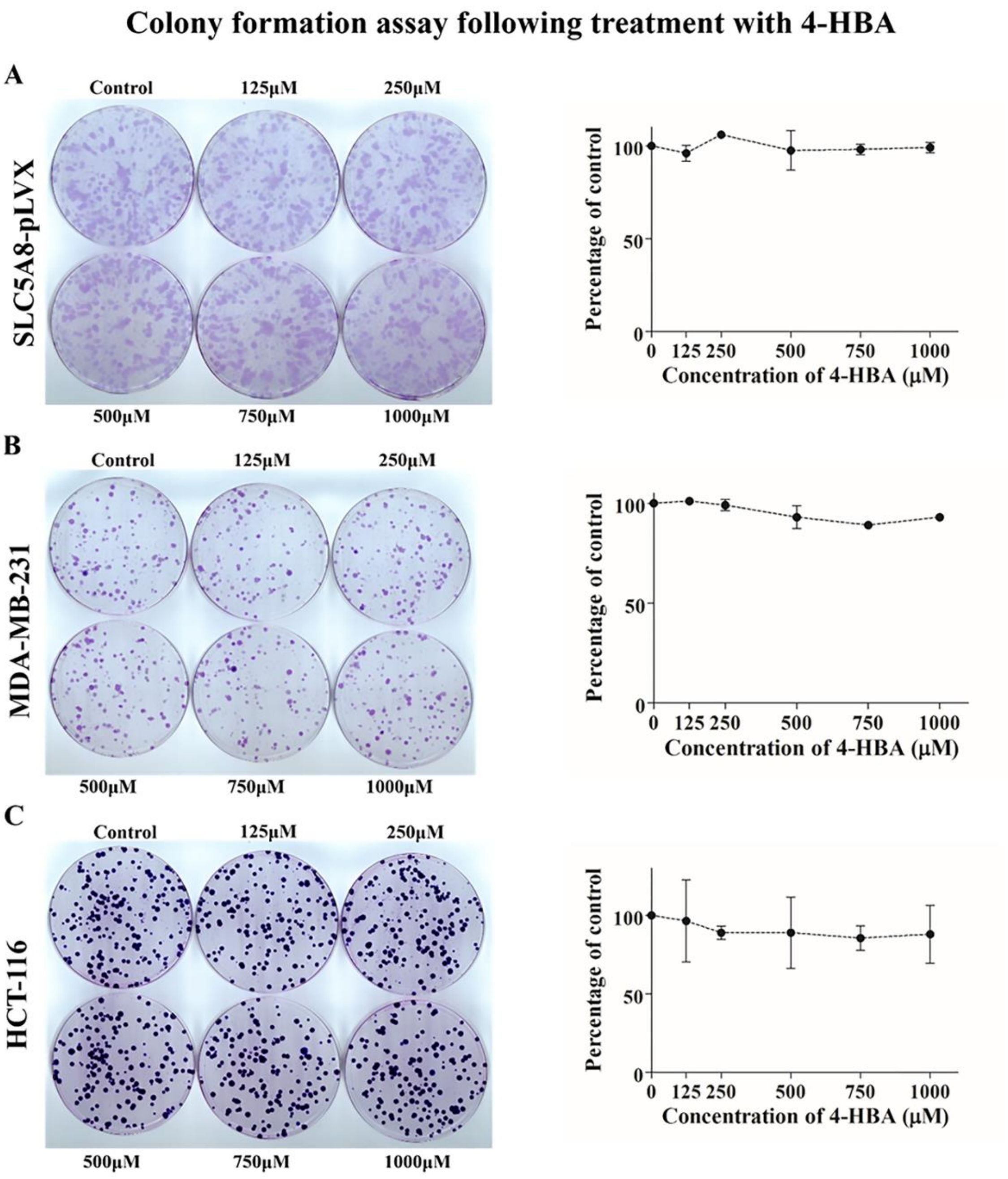

Figure S7. Effect of different concentrations of 4-HBA on colony formation in SLC5A8-PLVX (**A**), MDA-MB-231 (**B**), and HCT-116 (**C**) cells.

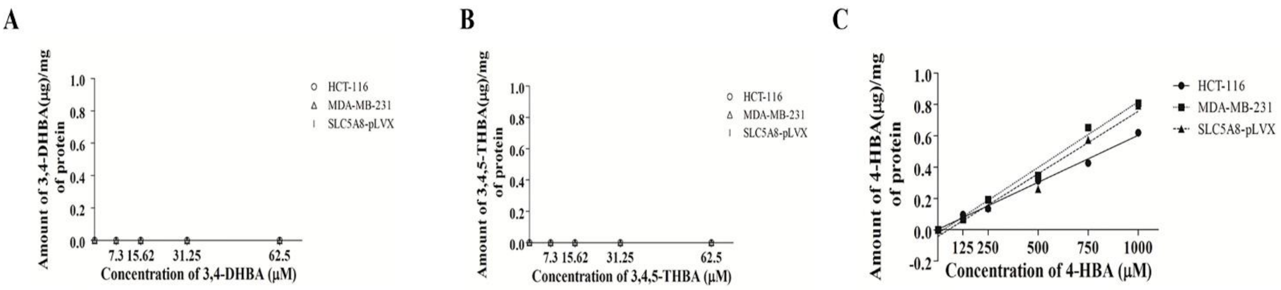

Figure S8. HPLC analysis showing the cellular uptake in SLC5A8-pLVX, MDA-MB-231 and HCT-116 cells following incubation with 3,4-DHBA (**A**), 3,4,5-THBA (**B**) and 4-HBA (**C**) in the cytosol.
